# Risk factors and prediction model for post-endoscopic lithotripsy infections

**DOI:** 10.3389/fsurg.2026.1861286

**Published:** 2026-10-01

**Authors:** Xiaotao Liang, Ziyong Lei, Yuxi Chen, Wei Fang, Ruming Liu, Xiaoyu Liu, Yizhe Yang, Lianhong Gao, Yongming Jiang

**Affiliations:** The Second Affiliated Hospital of Kunming Medical University, Yongchuan District People’s Hospital, Chongqing, China

**Keywords:** percutaneous nephrolithotomy, predictive model, systemic inflammatory response, ureteroscopic lithotripsy, urosepsis

## Abstract

**Objective:**

To identify independent risk factors for infectious complications (fever, SIRS, sepsis) after endoscopic lithotripsy and construct a multi-outcome hierarchical prediction model.

**Methods:**

Retrospective study of 914 patients (*n* = 914). Primary analysis used multinomial logistic regression for sepsis; sensitivity analysis used binary logistic regression for fever and SIRS with backward elimination. Bootstrap validation, AUC, calibration (MAE), and DCA were performed.

**Results:**

Primary analysis: positive urine culture (OR = 3.36), previous surgery (OR = 3.62), radiological signs of infection (OR = 4.02), and combined surgery (OR = 4.95) were independent risk factors for sepsis (AUC=0.794, MAE=0.008). Fever model: radiological signs and surgical modality (AUC=0.715). SIRS model: radiological signs and ULE+++ (OR = 5.1, AUC=0.73). DCA showed net clinical benefit for all three models.

**Conclusion:**

The multi-outcome hierarchical model effectively predicts postoperative infection risk. Nomograms guide individualized perioperative interventions.

## 1 Introduction

Urinary tract stones affect 13% in North America, 5%–9% in Europe, and 1%–5% in Asia (1). Ureteroscopic lithotripsy (URL) and percutaneous nephrolithotomy (PCNL) are standard treatments (2–4). Postoperative sepsis occurs in 0.9–4.7% (5)and is life-threatening. Preoperative diagnosis relies on urinalysis and urine culture, which have limited sensitivity (6–8). Previous studies identified risk factors for fever and sepsis, but most focused on single outcomes or single categories. Patient history (e.g., previous urological surgery) and renal imaging signs of infection have been underexplored. This study aimed to construct a multi-dimensional, multi-outcome prediction model integrating laboratory, imaging, host, and surgical factors.

## 2 Materials and methods

### 2.1 Materials

Study Population. From September 2023 to September 2024, 1,048 patients underwent endoscopic lithotripsy; 914 met inclusion criteria. Exclusion criteria: sepsis from other systems, long-term preoperative antibiotics/immunosuppressants, severe other infections, incomplete key variables. Patients with pre-existing nephrostomy tubes, indwelling ureteral stents, or congenital/acquired renal abnormalities were not excluded from the study. In this retrospective analysis, these conditions were not consistently recorded in all patients' medical records and could not be reliably analyzed as separate variables. However, including these patients reflects the real-world clinical population undergoing endoscopic lithotripsy and enhances the generalizability of our findings. We acknowledge this as a limitation and have added it to the Discussion section.

Diagnostic Criteria. Fever: postoperative temperature >38 °C without other SIRS criteria. SIRS: ≥2 of: WBC >12 or <4 × 10⁹/L or >10% bands; temperature >38 °C or <36 °C; heart rate >90/min; respiratory rate >20/min or PaCO₂ < 32 mmHg (9). Sepsis: urinary tract infection plus SIRS (2019 EAU guidelines) (10).

### 2.2 Methods

#### 2.2.1 Data collection

The data were sourced from patients diagnosed with calculi who underwent minimally invasive endoscopic lithotripsy in Ward 4 of the Department of Urology at the Second Affiliated Hospital of Kunming Medical University, primarily encompassing the following variables. Variables: demographics, medical history, preoperative antibiotics(Preoperative antibiotic use was defined as the administration of antibiotics within 7 days prior to surgery, with duration ranging from a single prophylactic dose to a full therapeutic course depending on clinical indication.), urinalysis [leukocytes, nitrite, ULE graded(-/+/++/+++), bacterial count, culture], blood tests (WBC, neutrophils, lymphocytes, hemoglobin, albumin, creatinine, UA, glucose, IL-6), imaging (stone length, CT value, radiological signs of infection), surgical modality, operative time.

#### 2.2.2 Definitions

BMI: underweight <18.5, normal 18.5–24, overweight ≥24 kg/m^2^. Diabetes: FPG ≥7.0 mmol/L, 2 h PG ≥11.1 mmol/L, or random glucose ≥11.1 mmol/L with symptoms (11). Radiological signs of infection were defined based on preoperative non-contrast CT, adapted from established criteria. Patients were classified as having radiological signs of infection if they presented with any of the following findings: Stone-related signs: (1) low-density complete staghorn calculi; (2) heterogeneous stone density; (3) imbricated or laminated stone morphology. Renal parenchymal and perirenal signs: (4) perirenal or renal sinus fat stranding; (5) irregular renal contour; (6) non-dilated thinning of the renal parenchyma; (7) thickening of the renal fascia. The presence of any one of these findings on preoperative CT qualified the patient as having “radiological signs of infection.” History of previous urological surgery was recorded as a binary variable (present vs. absent). Although the specific type of prior surgery (e.g., PCNL, URS, open surgery) was recorded in some cases, these procedures were performed at various outside hospitals with incomplete and inconsistent documentation. We were therefore unable to reliably categorize patients by the specific type or number of prior surgeries. All prior ipsilateral urological procedures were combined into a single binary category, as any prior surgical intervention may alter the local anatomy, cause scarring, and predispose to bacterial colonization, regardless of the specific procedure type. Combined surgery: single-stage URL + PCNL.

#### 2.2.3 Statistical analysis

Outcomes were multinomial unordered categorical (0 = no complication, 1 = fever, 2 = SIRS, 3 = sepsis). SPSS 27.0 and R 4.4.1 were used. Univariate: Kruskal–Wallis H for continuous variables [median (IQR)], chi-square/Fisher for categorical. Collinearity: VIF >5 considered significant. Primary analysis (sepsis): multinomial logistic regression with 4 core variables. Following the “10 events per variable” rule of thumb, we restricted the number of predictors to four to minimize overfitting, as only 32 sepsis events were available (approximately 8 events per predictor). We acknowledge that this ratio is slightly below the recommended threshold, and therefore interpret the results with appropriate caution. Sensitivity analysis (fever and SIRS): binary logistic regression with backward elimination (*P* > 0.05 for removal) from 9 candidate variables. The purpose of this sensitivity analysis was to explore outcome-specific risk factors for fever and SIRS separately, rather than to validate the primary analysis. To assess the robustness of variable selection, we additionally performed LASSO regression with 10-fold cross-validation using the R package glmnet. The optimal penalty parameter *λ* was selected as the value yielding minimum cross-validated deviance, and variables with non-zero coefficients at the optimal *λ* were retained. The results from both methods were compared (Supplementary Table S1). Given the exploratory nature of this analysis and the limited sample size (fever: 117 events; SIRS: 82 events), findings from the sensitivity analysis are presented with appropriate caution. Model validation: All models were internally validated using bootstrap resampling (1,000 iterations). The procedure was: (1) a bootstrap sample was drawn with replacement from the original dataset; (2) the model was refit in the bootstrap sample and AUC was calculated; (3) the optimism was estimated as the difference between the AUC in the original dataset and the average AUC across all bootstrap samples; (4) the bias-corrected AUC was obtained by subtracting the optimism from the original AUC. Calibration was assessed by plotting calibration curves based on bootstrap samples and quantified using the mean absolute error (MAE). Clinical utility was evaluated using decision curve analysis (DCA). No training/test split was performed, in order to preserve maximum sample size given the limited number of sepsis events.

## 3 Results

Patient Characteristics. Of 914 patients, 683 (74.7%) had no complications, 117 (12.8%) fever, 82 (9.0%) SIRS, 32 (3.5%) sepsis. Significant differences were observed for sex, surgical modality, and history of previous surgery (*P* < 0.05).

### 3.1 Univariate analysis

Significant markers (*P* < 0.05): urine leukocytes, urine culture, nitrite, ULE, IL-6, urine bacterial count, hemoglobin, albumin, stone length, radiological signs of infection, preoperative antibiotic use, operative time. Non-significant: urine pH, WBC, neutrophils, lymphocytes, platelets, creatinine, UA, glucose. Notably, patients receiving preoperative antibiotics had higher rates of fever, SIRS, and sepsis, which likely reflects that antibiotics were preferentially prescribed to patients already identified as high-risk for infection (e.g., those with positive urine cultures or other risk factors), rather than indicating a causal relationship.

### 3.2 Collinearity diagnosis

High collinearity was found between IL-6 and hemoglobin (variance proportion 0.90) and albumin (0.93), and between urine bacterial count and urine culture (0.91). Hemoglobin, albumin, and urine bacterial count were removed.

### 3.3 Multivariable logistic regression

#### 3.3.1 Primary analysis (sepsis)

Positive urine culture (OR = 3.356, 95%CI:1.579-7.135), previous surgery (OR = 3.622, 95%CI:1.616–8.121), radiological signs of infection (OR = 4.020, 95%CI:1.734–9.319), and combined surgery (OR = 4.945, 95%CI:1.546–15.824) were independent risk factors for sepsis.

#### 3.3.2 Sensitivity analysis

##### 3.3.2.1 Fever model

Retained radiological signs of infection (OR = 2.66) and PCNL (OR = 4.36) and combined surgery (OR = 4.03) (all *P* < 0.001).

##### 3.3.2.2 SIRS model

Retained radiological signs of infection (OR = 2.66) and ULE “+++” (OR = 5.1, *P* = 0.007). Surgical modality was not retained.

### 3.4 Nomograms

Given the limited number of sepsis cases (*n* = 32), the primary analysis was necessarily focused on constructing a reliable predictive model for this most severe outcome. Accordingly, a nomogram based on the primary multinomial logistic regression model was developed to estimate the risk of sepsis (Figure 1). In this nomogram, the contribution of each independent risk factor to the total risk score is represented by the length of the corresponding line segment: positive urine culture contributed the highest score (99 points), followed by history of previous surgery (96 points), radiological signs of renal infection (92 points), and combined surgery (85 points). The total point scale ranges from 0 to 400, and the predicted probability of sepsis can be read directly from the bottom axis.

**Figure 1:**
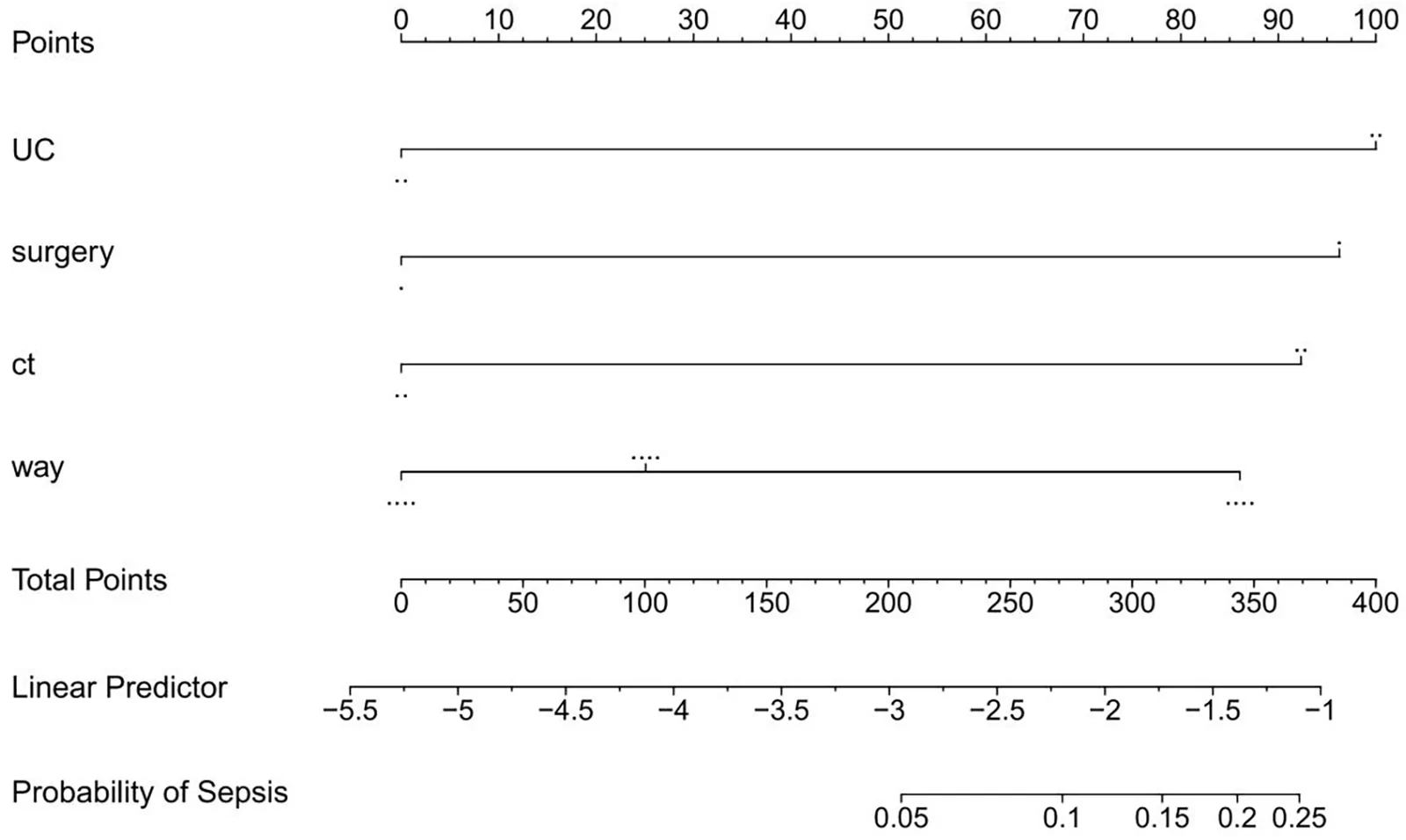
Main analysis nomogram of sepsis.

For the other two infectious outcomes—fever and systemic inflammatory response syndrome (SIRS)—the predictive models were derived from sensitivity analyses using independent binary logistic regression, and the corresponding nomograms are presented separately (Figures 2–1, 2–2). The fever nomogram retained radiological signs of renal infection (66 points) and surgical modality (PCNL: 93 points; combined surgery: 99 points). The total point scale for fever ranges from 0 to 180. The SIRS nomogram retained radiological signs of renal infection (56 points) and urinary leukocyte esterase (ULE) grade “+++” (99 points), with a total point scale also ranging from 0 to 180. These sensitivity-based nomograms complement the primary sepsis nomogram and provide clinicians with practical tools for individualized risk assessment across the entire spectrum of postoperative infectious complications.

**Figure 2:**
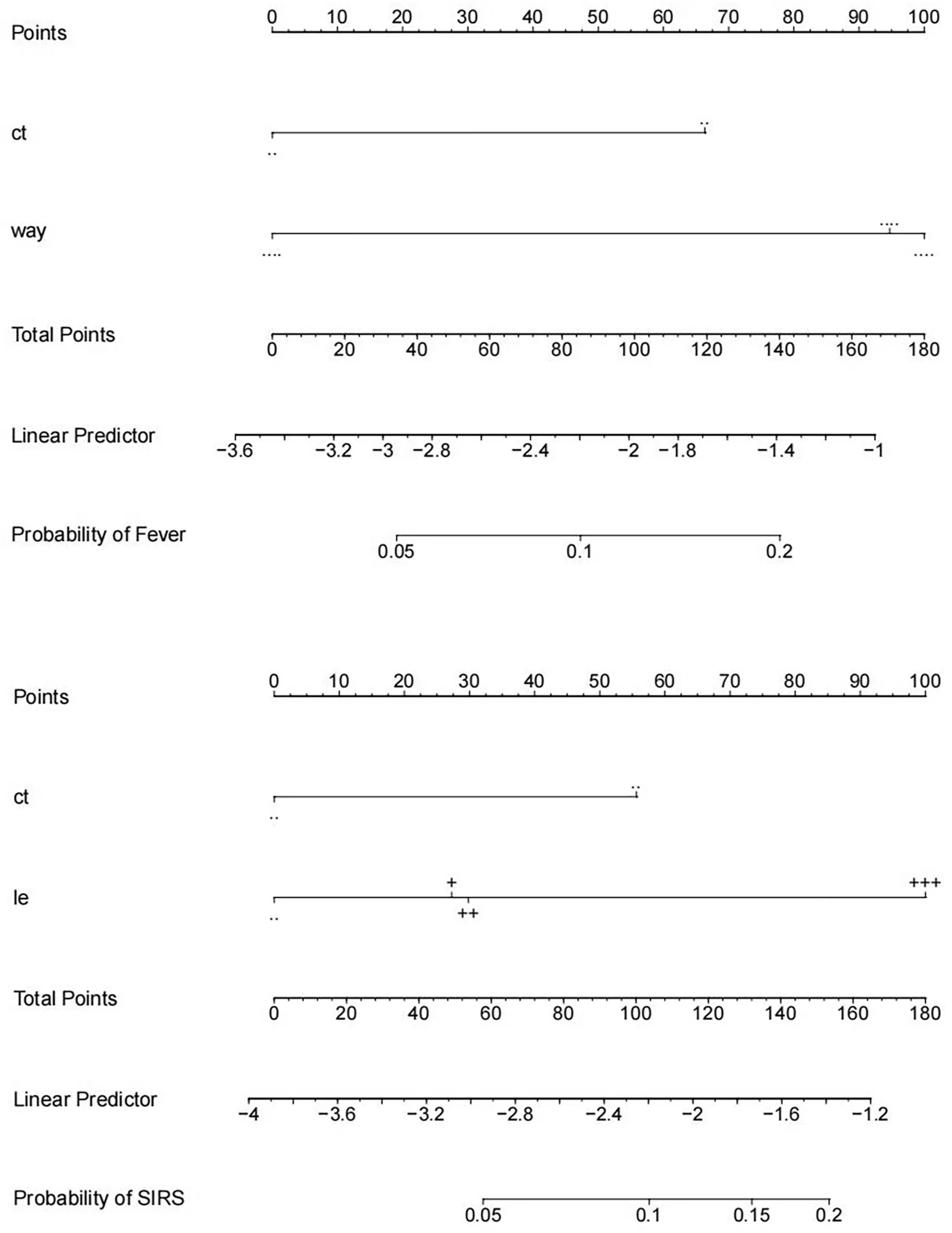
Sensitivity analysis fever and SIRS line chart.

Clinical application of the nomogram: The nomogram is used as follows: (1) locate each patient's value for each predictor on the corresponding axis; (2) draw a vertical line upward to the “Points” axis to obtain the score for each variable; (3) sum all individual scores to obtain the Total Points; (4) draw a vertical line downward from the Total Points axis to the “Probability of Sepsis” axis to read the predicted probability.

Example: A patient with positive urine culture (99 points), history of previous surgery (96 points), radiological signs of infection (92 points), and undergoing combined surgery (85 points) has a total score of 372 points, corresponding to a predicted sepsis probability of approximately 23%. This identifies the patient as high-risk, warranting enhanced perioperative monitoring and prophylactic interventions.

### 3.5 Model performance

Sepsis model: AUC=0.794 (bias-corrected 0.769), MAE=0.008, R^2^ = 0.997; DCA net benefit up to threshold ∼0.25. Given the low incidence of sepsis (32/914, 3.5%), we additionally report metrics suitable for imbalanced data. At the optimal threshold determined by the Youden index, the model achieved a sensitivity of 84.4%, a specificity of 67.7%, a positive predictive value (PPV) of 8.7%, and a negative predictive value (NPV) of 99.2%. The F1-score was 0.157, and the precision-recall AUC (PR-AUC) was 0.124, which is substantially higher than the random baseline of 0.035 (the event rate), confirming the model's predictive utility in this highly imbalanced setting. The high NPV (99.2%) indicates that the model can reliably rule out sepsis in low-risk patients. Fever model: AUC=0.715 (corrected 0.709), MAE=0.005; DCA net benefit for thresholds <0.28. SIRS model: AUC=0.73 (corrected 0.72), MAE=0.013; DCA net benefit for thresholds <0.31 (Figure 3–7).

**Figure 3:**
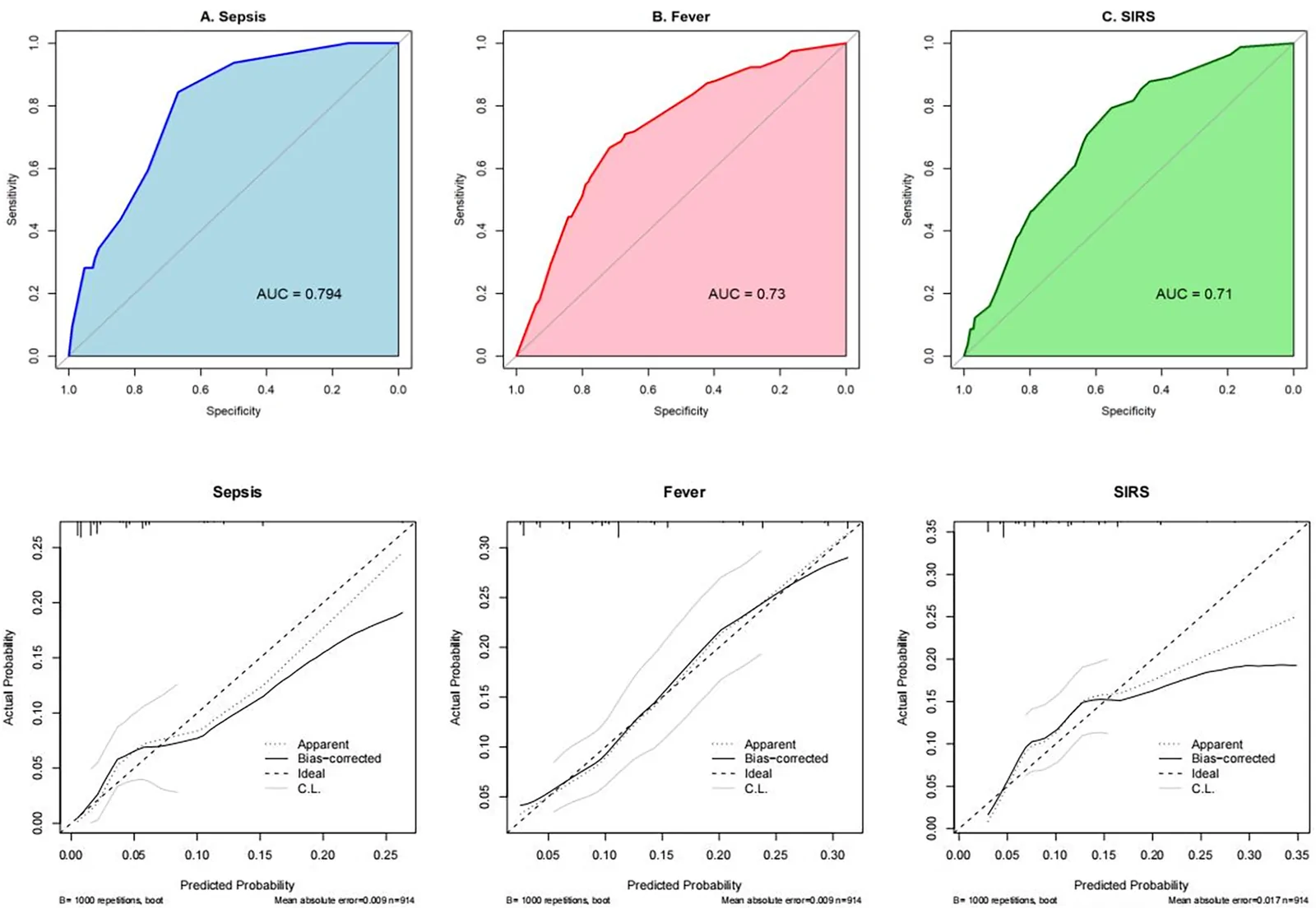
Main analysis ROC curve and main analysis calibration curve.

**Figure 4:**
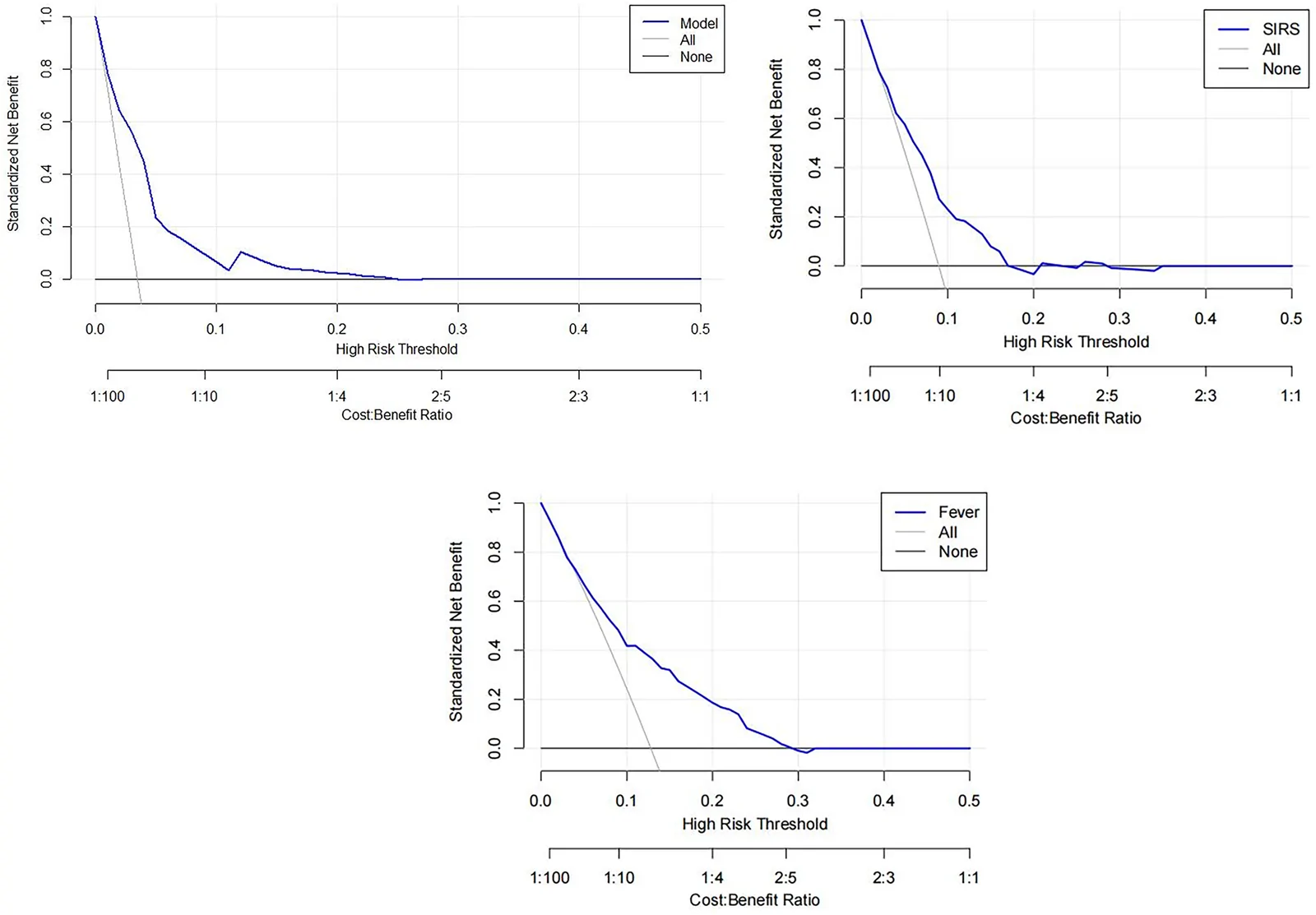
Main analysis decision curve.

**Figure 5:**
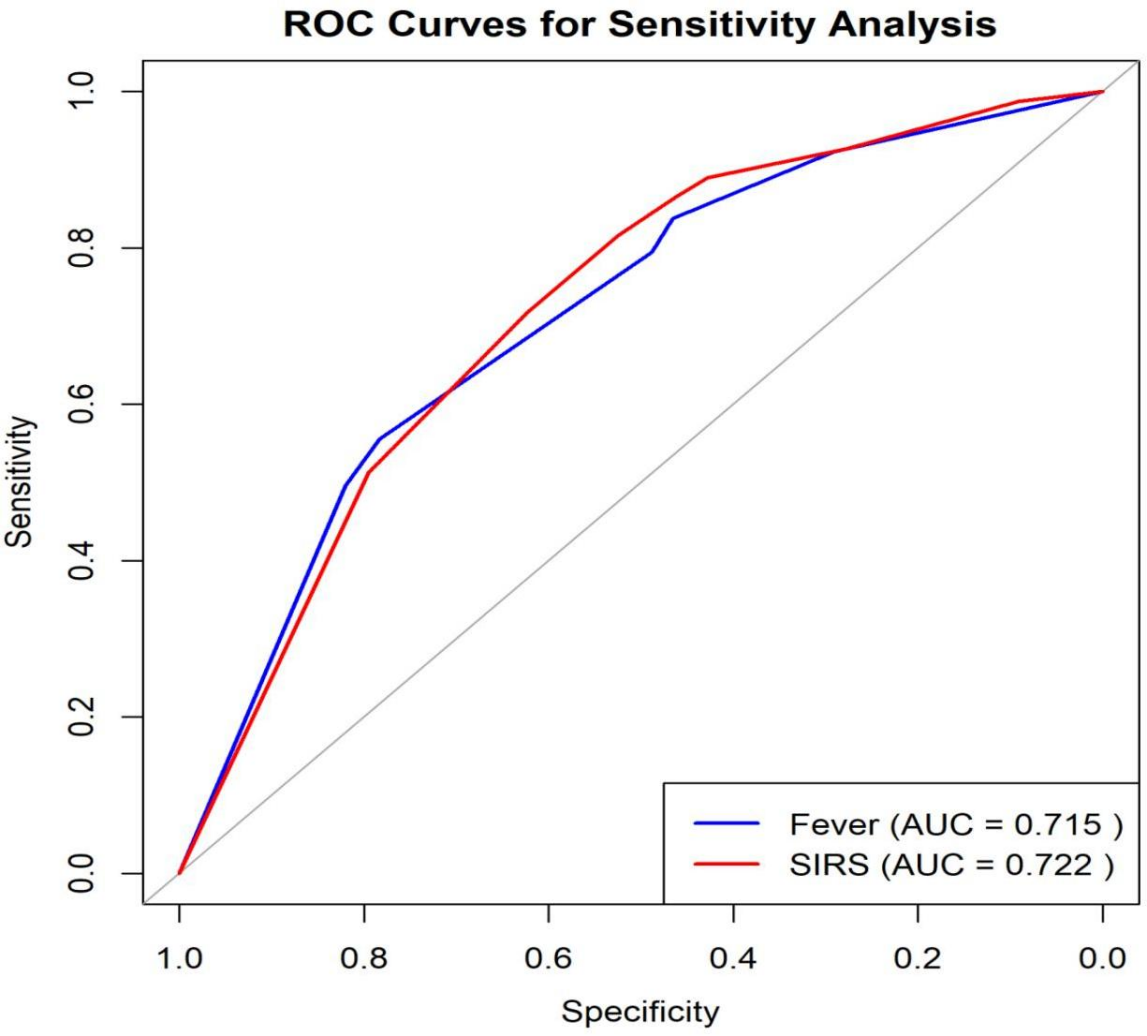
Sensitivity analysis of fever, SIRS ROC curve.

**Figure 6:**
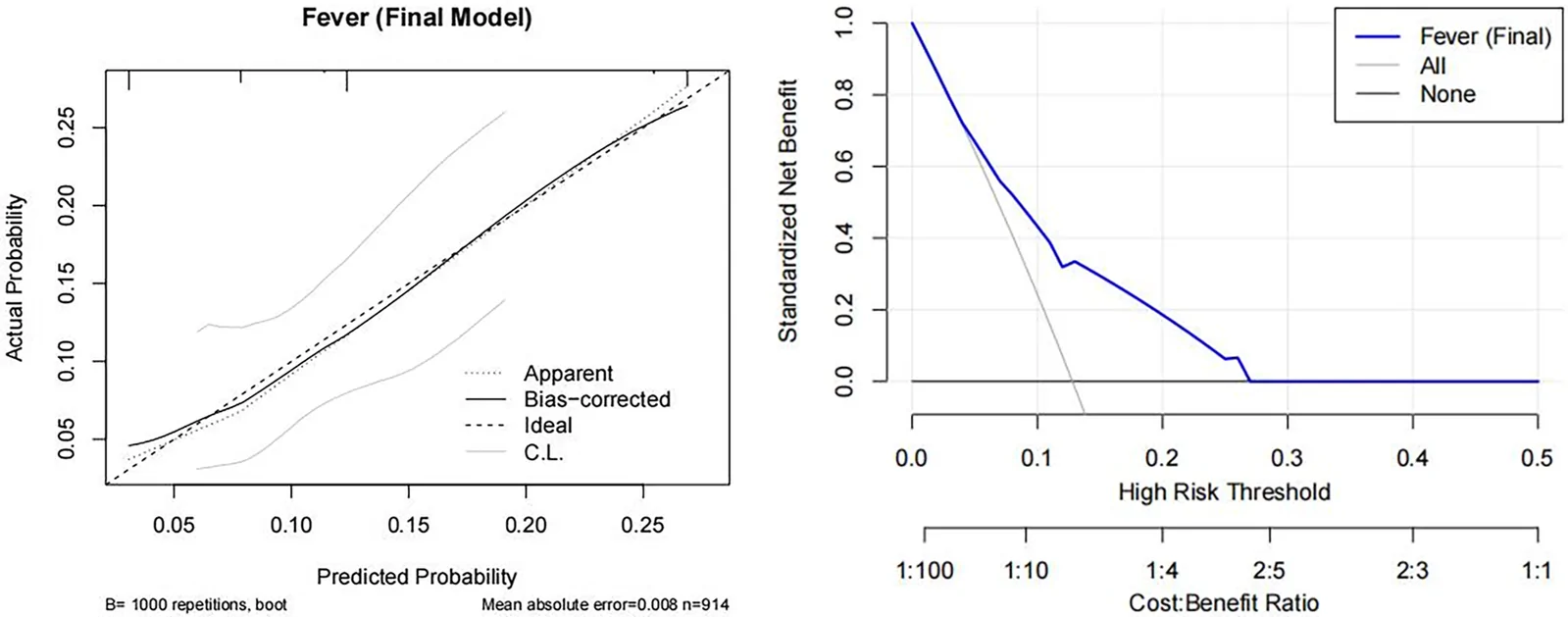
Calibration curve and decision curve for fever in the sensitivity analysis.

**Figure 7:**
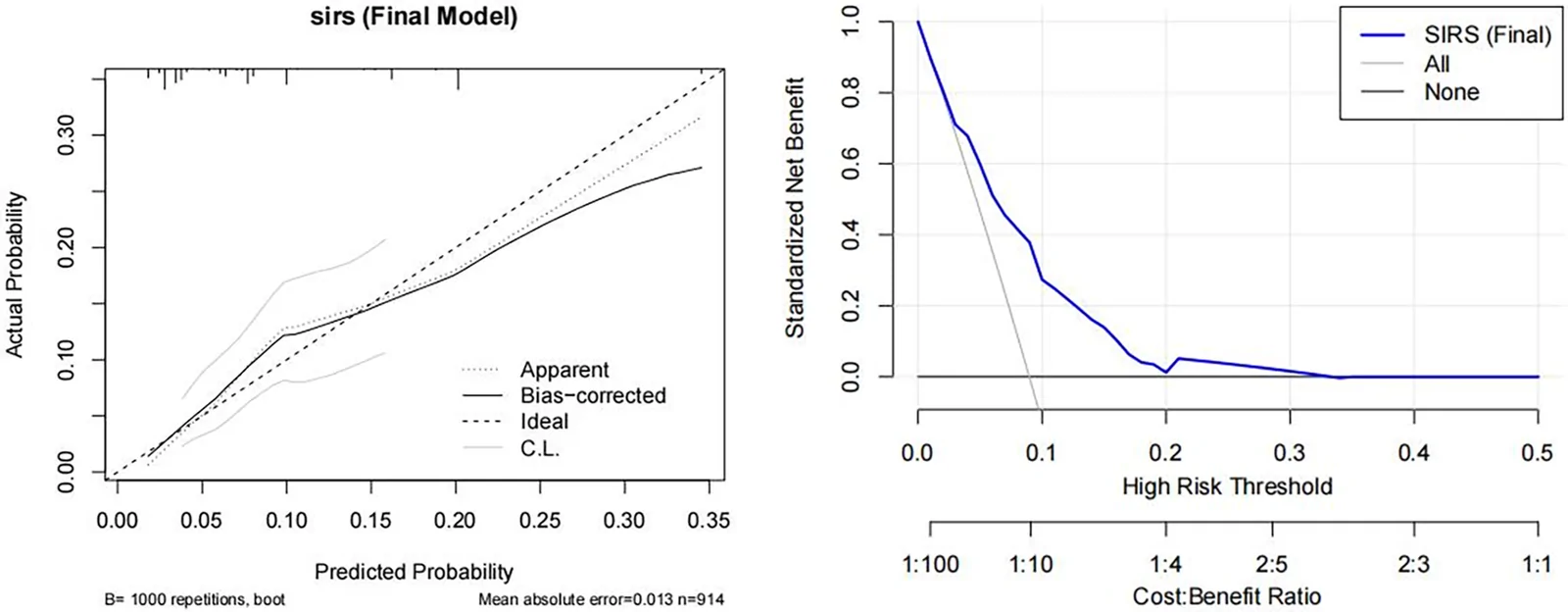
The calibration curve and decision curve of SIRS in the sensitivity analysis.

## 4 Discussion

Endoscopic lithotripsy is the preferred treatment for urinary stones due to minimal invasiveness (12–14). However, complications such as bleeding, fever, SIRS, and sepsis remain concerns (15–17). Urosepsis has a mortality rate of 30%–40% (18, 19). Most previous studies treated fever, SIRS, and sepsis as a continuum, potentially missing distinct risk factor profiles. Our study supports considering them as distinct complications. For example, positive urine culture was much stronger for sepsis than for fever, while ULE was significant only for SIRS. This underscores the value of multinomial unordered analysis. By integrating imaging, patient history, and surgical factors with laboratory markers, our study provides a more holistic approach.

### 4.1 Primary analysis

#### 4.1.1 Urine culture

Positive urine culture was most strongly associated with sepsis (OR = 3.36)but only weakly (SIRS) or non-significantly (fever) with milder complications. This heterogeneity would have been masked if outcomes were treated as ordinal. The strong correlation between this urine culture and sepsis is consistent with previous studies (20, 21). In our cohort, E. coli was the most common pathogen (41.7%) (22, 23). However, 40.6% of sepsis patients had negative preoperative urine cultures, highlighting the limitation of relying solely on this marker. Recent studies suggest that renal pelvic or stone cultures may have greater predictive accuracy for postoperative sepsis than midstream urine cultures (24).It is noteworthy that preoperative antibiotic use was associated with higher rates of infectious complications in univariate analysis, which likely reflects indication bias—patients with suspected or confirmed preoperative infection were more likely to receive antibiotics and were also inherently at higher risk for postoperative infectious complications. This finding carries important clinical implications. The fact that 40.6% of sepsis patients had negative preoperative urine cultures suggests that urine culture alone is insufficient for preoperative risk stratification. Relying solely on this test would miss a substantial proportion of high-risk patients. Therefore, for complex stone patients, we recommend a broader preoperative workup that integrates: (1) imaging assessment for radiological signs of infection (e.g., low-density staghorn calculi, perirenal fat stranding); (2) careful history taking for prior urological surgery; and (3) comprehensive laboratory evaluation including inflammatory markers. This multi-dimensional approach may improve the identification of high-risk patients who would benefit from enhanced perioperative monitoring and prophylactic interventions, even in the absence of a positive urine culture.

#### 4.1.2 Surgical modality

Combined surgery (URL + PCNL) had the strongest association with sepsis (OR = 4.95), reflecting surgical complexity and prolonged operative time. This finding suggests that for complex stones requiring single-stage clearance, the risk of sepsis must be carefully weighed (25).

#### 4.1.3 Radiological signs of infection

This was a strong independent risk factor for sepsis (OR = 4.02), consistent with previous reports (26). The high point contribution in the nomogram (92 points) supports its central role in risk stratification.

#### 4.1.4 History of previous surgery

This was an independent risk factor for sepsis (OR = 3.62) (27). Although some studies have not found this association (28), our results align with EAU guidelines recommending thorough infection screening for these patients (29).

These four factors -microbiology (urine culture), host (surgical history), imaging (radiological signs), and surgical trauma (combined surgery) – each contributed high points in the sepsis nomogram (99, 96, 92, and 85 respectively). Their combined use allows multi-dimensional risk profiling, which is more informative than any single indicator.

### 4.2 Sensitivity analysis

The fever model confirmed that radiological signs and surgical modality are core drivers of fever. The SIRS model revealed that ULE “+++” was the dominant predictor (OR = 5.1), and surgical modality was not retained, suggesting that the effect of surgical trauma on SIRS is mediated by local inflammation (ULE). This provides statistical validation of the “trauma-inflammation-SIRS' pathway (30).

### 4.3 Clinical implications

Patients with positive urine culture and previous surgery warrant heightened vigilance for sepsis. Patients with radiological signs of infection undergoing combined surgery are at highest risk for fever. Patients with ULE “+++” should be monitored for SIRS regardless of urine culture results. Patients with none of these risk factors can be managed routinely.

### 4.4 Limitations

This study has several limitations. (1) The small sample size for sepsis (*n* = 32) limited the number of variables that could be included in the primary analysis. Although we followed the “10 events per variable” rule and restricted the model to four predictors (approximately 8 events per predictor), this ratio is slightly below the recommended threshold and may increase the risk of overfitting. Bootstrap internal validation showed minimal optimism, but this cannot fully compensate for the limited sample size. External validation in an independent cohort is essential before clinical application. (2) The low PPV (8.7%) reflects the inherent challenge of predicting rare events; positive predictions should be interpreted with caution and combined with other clinical information. (3) The inconsistency between LASSO and backward elimination in the sensitivity analysis—LASSO retaining stone_size for fever (vs. ct and way by backward elimination) and retaining no variables for SIRS (vs. ct and LE by backward elimination)—highlights the challenges of variable selection in small samples. LASSO's failure to retain variables in the SIRS model likely reflects the conservative nature of the penalty under limited sample size (82 events), rather than an absence of predictive signals. These findings underscore the exploratory nature of our analyses and the urgent need for external validation in larger cohorts. (4) Single-center retrospective design may introduce bias. (5) Unmeasured confounders (e.g., specific antibiotic regimens) may exist. The multi-outcome hierarchical prediction model system effectively assesses postoperative infection risk after endoscopic lithotripsy. Positive urine culture and previous surgery are specific early warning indicators for sepsis. Radiological signs of infection are relevant across the entire spectrum of complications. Surgical modality predominantly influences fever risk, while ULE is a key predictor for SIRS. The nomograms provide a practical tool for individualized risk assessment and perioperative stratified interventions. In addition, the specific type, dose, and duration of preoperative antibiotic regimens were not consistently recorded for all patients, and therefore could not be included as separate variables in the analysis.

Additionally, our prediction model was developed and internally validated using a single-center dataset. External validation in an independent, preferably multi-center cohort is essential before the model can be confidently applied in routine clinical practice. Future studies should prospectively validate the model's performance, calibration, and clinical utility across diverse patient populations and clinical settings.

## Data Availability

The raw data supporting the conclusions of this article will be made available by the authors, without undue reservation.
