# Supplementary material for: Risk factors and prediction model for post-endoscopic lithotripsy infections
Source: Front Surg. 2026 Oct 1;13:1861286. doi: 10.3389/fsurg.2026.1861286 (PMC13669194; doi:10.3389/fsurg.2026.1861286)
Supplement: Supplementary file 1 [file Datasheet1.pdf]

Outcome	Method	Variables_Retained
Fever	Backward elimination	ct, way
Fever	LASSO	size
SIRS	Backward elimination	ct, LE
SIRS	LASSO	

Metric	Value
Sensitivi	0.844
Specifici	0.677
PPV	0.087
NPV	0.992
F1	0.157
