# Supplementary material for: Risk factors and prediction model for post-endoscopic lithotripsy infections
Source: Front Surg. 2026 Oct 1;13:1861286. doi: 10.3389/fsurg.2026.1861286 (PMC13669194; doi:10.3389/fsurg.2026.1861286)
Supplement: Supplementary file 2 [file Datasheet2.pdf]

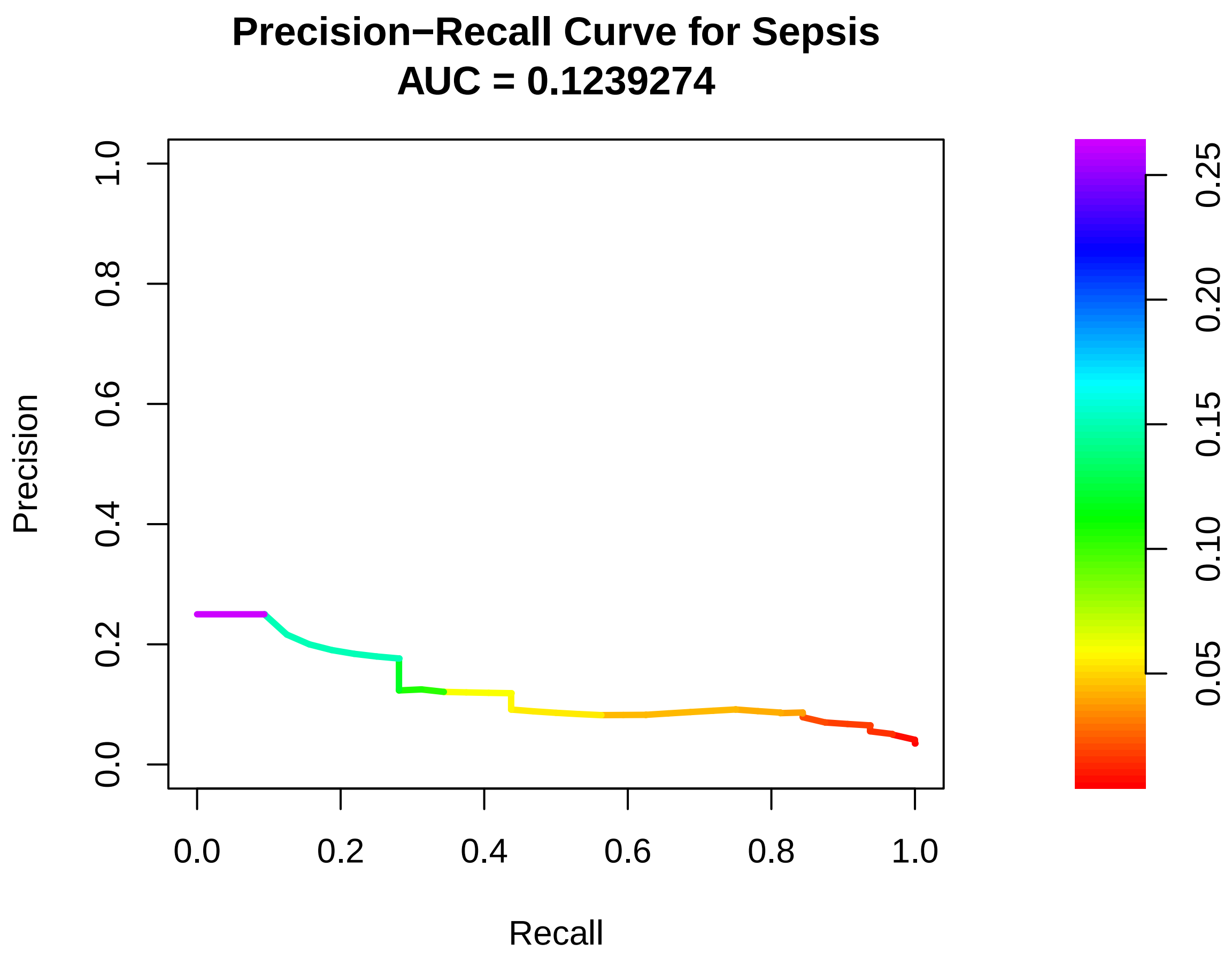
Precision−Recall Curve for Sepsis
AUC = 0.1239274
Precision
Recall
0.0
0.2
0.4
0.6
0.8
1.0
0.05
0.10
0.15
0.20
0.25
